# The Impact of a Prolonged Multivitamin Shortage on Home Parenteral Nutrition Patients: A Single-Center Retrospective Cohort Study with Case Reports of Wernicke’s Encephalopathy

**DOI:** 10.3390/nu17091500

**Published:** 2025-04-29

**Authors:** Chanita Unhapipatpong, Natalie C. Lam, Christopher Wang, Katherine J. P. Schwenger, Celeste Arca, Ka-Wai Chin, Ann MacGillivray, Clement Yuen, Ian Pang, Johane P. Allard

**Affiliations:** 1 Division of Gastroenterology, Department of Medicine, Toronto General Hospital, University of Toronto, Toronto, ON M5G 2C4, Canada; chanita@kkumail.com (C.U.); nataliecarmenlam@gmail.com (N.C.L.); katherine.schwenger@uhn.ca (K.J.P.S.); celeste.arca@uhn.ca (C.A.); ka-wai.chin@uhn.ca (K.-W.C.); ann.macgillivray@uhn.ca (A.M.); clement.yuen@uhn.ca (C.Y.); ian.pang@uhn.ca (I.P.); 2 Division of Nutrition, Department of Medicine, Khon Kaen Hospital, Khon Kaen 40000, Thailand; 3 Division of Gastroenterology, Department of Medicine, St. Joseph’s Health Centre, Toronto, ON M6R 1B5, Canada; christopherwang916@gmail.com

**Keywords:** parenteral nutrition, multivitamin shortage, Wernicke encephalopathy, micronutrient deficiency

## Abstract

**Background/Objectives**: Shortages in parenteral nutrition (PN) micronutrient components can lead to deficiencies in patients heavily relying on home PN (HPN) to meet nutritional requirements. Despite monitoring, this can cause severe and even life-threatening conditions if intravenous (IV) micronutrients are not available for a prolonged period. **Methods**: We conducted a retrospective study to evaluate the effect of an IV multivitamin shortage that occurred between December 2022 and July 2023. The study included patients at high risk for multivitamin deficiencies who received HPN for at least 5 days. Patients were classified into two groups: those compliant with instructions to take additional oral multivitamin supplements to compensate for the shortage and those who were not compliant. Monitoring included tracking symptoms and routine bloodwork, which measured certain vitamins, excluding thiamine. **Results**: A total of 25 HPN patients were identified. Among them, 56% (*n* = 14) were compliant with daily oral multivitamin supplementation. No significant differences in pre- and post-shortage bloodwork were observed, but there was a significant difference in bicarbonate changes between the compliant and non-compliant groups (0 (−0.9, 1) vs. −2 (−8, −1), *p* = 0.04, respectively). Approximately 68% of all patients reported new symptoms during the shortage, but no significant difference was observed between groups. Three patients known to have increased gastrointestinal losses (two compliant and one non-compliant) required hospitalization: two had Wernicke’s encephalopathy reversed with thiamine infusion. **Conclusions**: When IV multivitamins are unavailable for an extended period, at-risk patients need to be closely monitored by the HPN team, particularly for compliance to oral supplementation and for symptoms of thiamine deficiency when blood level monitoring is not feasible.

## 1. Introduction

Patients with type 3 intestinal failure who have a metabolically stable chronic condition of malabsorption or an inability to meet macronutrient, micronutrient, water, and electrolyte requirements may require home parenteral nutrition (HPN) to prevent malnutrition and dehydration and to sustain life [1,2,3,4,5,6]. Many countries across the Americas, Europe, and Asia have faced periodic shortages of PN components over the decades, including essential trace elements, multivitamins, and amino acids, making it a global issue [4,7,8,9,10,11,12,13]. One of the commercial multivitamin products, Multi-12 (Sandoz Canada Inc., Boucherville, QC, Canada), is used across HPN programs in Canada and delivered to patients from their HPN providers. The province of Ontario, Canada, experienced localized shortages of the intravenous (IV) multivitamin infusion [5]. The severity of shortages across HPN programs differed depending on their respective providers and their stocks. Many factors can contribute to shortages; however, in this case, it was the result of global supply chain issues, the reliance on a sole manufacturer, the lack of alternative supply for these IV multivitamins, and its allocation methods to different HPN programs in Canada.

To help manage the IV multivitamin shortage in adult PN patients, the American Society for Parenteral and Enteral Nutrition (ASPEN) issued a set of recommendations outlining strategies and prioritization guidance for institutions, including suggested dosing adjustments and risk-based allocation [14]. Although ASPEN also recommended institutions to administer individual parenteral vitamin entities such as thiamine, ascorbic acid, pyridoxine, and folic acid, this recommendation is often not possible to follow due to logistic issues, the availability of the specific products, or supply shortage issues [14]. Thus, closely monitoring HPN patients, particularly to ensure the adequacy of oral vitamins during the IV multivitamin shortage, is frequently the alternative.

We recently experienced a severe and prolonged shortage of IV multivitamins and decided to share our experience by conducting a study assessing certain vitamin levels, biochemical blood work, nutritional status, symptoms, and clinical outcomes of high-risk patients heavily relying on HPN to meet their requirements. These patients were instructed to take oral multivitamin supplements to compensate for the lack of IV multivitamins, and those who were compliant were compared to those who were not compliant with the instructions.

## 2. Materials and Methods

This single-center retrospective cohort study was conducted from December 2022 to July 2023 and received research ethics approval on 8 March 2023. Inclusion criteria were adult patients aged ≥ 18 years who were PN-dependent, defined as receiving HPN for at least 5 out of 7 days a week and having been on HPN for at least 4 months during the shortage period. Exclusion criteria were patients receiving PN for less than 5 days per week or missing medical records. All patients were treated according to the institution’s existing management protocols and standards of care. At this institution, it is standard practice to provide all home PN patients with 10 mL of IV multivitamin to be injected in the PN solution prior to infusion. The specific breakdown of IV multivitamin compared to the standard recommendation of IV multivitamin in HPN, oral multivitamin, and dietary reference intakes (DRIs) is included in Table S1 [15]. Informed consent was waived due to the retrospective/chart review nature of the study. All procedures performed in studies involving human participants were in accordance with the ethical standards of the institutional and/or national research committee and with the 1975 Helsinki Declaration and its later amendments or comparable ethical standards.

At this University Hospital, all recommendations were implemented to the best of the organization’s ability. The multivitamin shortage, initially predicted to be resolved by February 2023, began in December 2022. In response, the hospital followed ASPEN’s recommendations by rationing IV multivitamins based on patients’ dependence on parenteral nutrition (e.g., three times per week instead of daily) [14]. Based on our institutional dosing strategy, patients who previously received IV multivitamins in their PN 6 to 7 days per week were given 10 mL of IV multivitamins five days per week. Patients receiving HPN five days per week were given IV multivitamins three times per week, those on HPN four days per week received it twice per week, and patients receiving PN three days or fewer per week received IV multivitamins once per week. Patients were also advised to begin oral multivitamin supplementation immediately after the shortage commenced.

**Figure 1:**
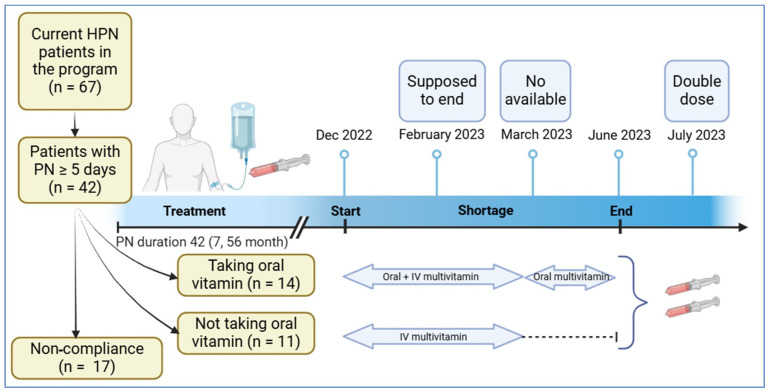
Timeline of Shortage and Patient Flow Diagram (IV multivitamins were given during the shortage based on the patient’s HPN reliance. Patients that previously received IV multivitamin in their PN 6–7 days a week were given 10 mL of IV multivitamin for five days a week. Patients receiving HPN 5 days a week were given IV multivitamin 3 days a week). Created in BioRender. Unhapipatpong, C. (2025) https://BioRender.com/r97u682 (accessed on 20 February 2025).

It is challenging to predict the duration of an IV multivitamin shortage. By March 2023, the shortage intensified, preventing the addition of IV multivitamins to HPN prescriptions. The hospital then adopted ASPEN’s recommendation, instructing all patients about the importance of taking daily oral multivitamins [16]. The HPN team reviewed patient files to ensure feasibility, contacted patients by phone, and provided guidance on specific over-the-counter oral multivitamins that closely matched IV formulations or were locally available (see Supplementary Table S1). Throughout the shortage, patients were monitored per standard practice with bloodwork, which included certain vitamins, performed every 3 to 6 months, depending on clinical stability and HPN requirements. When the shortage ended in June 2023, all patients were notified. To support repletion of tissue stores, the IV multivitamin dosage was increased from 10 mL to 20 mL in HPN prescriptions for one month, starting in mid-July 2023, based on expert opinion and feasibility (Figure 1), before resuming standard dosing.

For the retrospective study, we conducted a survey via telephone to assess compliance with oral multivitamin supplements and symptoms of vitamin deficiencies (Data S1). Since the multivitamins used by patients in our study varied in both dosage and formulation, direct comparisons of pills and dosages were not feasible. Therefore, we defined the compliant group as those who consumed at least 70% of the recommended dose based on each product’s specific formulation, which corresponded to taking the supplement an average of at least five days per week. Patients who took the supplement less than five days per week were classified as non-compliant. We also assessed various bloodwork parameters at pre- and post-shortage timepoints. Data were collected by one study coordinator and included clinical, biochemical, and PN parameters as well as complications and hospitalizations related or unrelated to the consumption of multivitamin tablets and/or multivitamin deficiencies. Bloodwork results and self-reported symptoms from the survey were compared between the compliant and non-compliant groups at both the beginning and the end of the multivitamin shortage, prior to the resumption of IV multivitamin supplementation.

For statistical purposes, continuous variables were expressed as median (1st quartile and 3rd quartile), and categorical variables were expressed as absolute value (percentage); Kruskal–Wallis test was used for continuous variables and Fisher’s exact tests for categorical variables. Statistical tests were considered significant if *p* < 0.05. All statistical analyses were conducted using SAS version 9.4 program (SAS Institute, Cary, NC, USA).

## 3. Results

### 3.1. Results from Retrospective Cohort Study

There were 67 HPN patients, of which 25 met the inclusion criteria (see Figure 1). Out of the 25 individuals who were instructed to take a daily oral multivitamin supplement, only 14 (56%) complied. At the start of the IV multivitamin shortage, those who were compliant with daily oral multivitamin supplement were significantly older (median age of 64 versus 43 years) and were on significantly longer duration of PN (median of 52.5 versus 9 months of PN) compared to those who were not compliant. The laboratory test results were generally similar between groups except for significantly lower blood glucose (median 5.15 versus 6.00 mmol/L) and platelets (median 182 versus 294.5 × 10^9^/L) in the compliant versus non-compliant group (see Table 1). All other variables were not significantly different between groups. Reasons for not taking the recommended vitamin supplement in the non-compliant group (*n* = 11, 44%) included financial difficulties to afford the supplements, being forgetful, and inconsistent use.

Overall, during the shortage, we found no significant differences in anthropometrics or PN prescriptions (Table S2), reported symptoms, number of hospitalizations, or diagnosed deficiencies between the two groups (see Table 2). The most common symptoms reported in both groups were decreased energy level and appetite. Other reported symptoms included nausea, constipation, blurry vision, gastrointestinal pain, diarrhea, appearing pale, puffy eyes, increased body temperature, decreased blood pressure, difficulty breathing, vomiting, and memory loss (see Table 2). There were also no significant changes in anthropometrics and PN prescription between the two groups (see Supplementary Table S2).

The impact of compliance at the end of the IV multivitamin shortage showed no significant differences between groups except for a significantly higher blood sodium (140 versus 135 mmol/L) and significantly lower INR (1.0 versus 1.2) in compliant versus noncompliant groups (see Table 3). All the other variables were not significantly different. Changes over the period showed a significantly greater increase in INR and greater decrease in bicarbonate in the non-compliant versus the compliant group (see Table 3).

### 3.2. Case Reports of Patients Who Required Hospitalization

There were three hospitalizations for patients with severe symptoms (two compliant and one non-compliant), two of which were related to the IV multivitamin shortage in compliant patients. Their clinical timelines are briefly outlined below. This constitutes 2/67 (2.98%) of the institution’s total HPN patients (Table 4).

Case 1 is a 59-year-old female on HPN seven days a week due to a high-output entero-vaginal fistula. To manage her fistula output, she was advised to remain NPO, with minimal oral intake limited to clear fluids and medications. She consistently took oral multivitamins (compliant), which met daily B vitamin requirements (Table S1). Due to a shortage, her IV multivitamins were discontinued, and two months later, she developed nausea and dizziness during PN infusions. These symptoms persisted despite PN modifications, prompting her to seek medical attention, and she was diagnosed with a severe urinary tract infection and admitted for IV antibiotics. Her nausea and dizziness worsened, leading to a brain magnetic resonance imaging (MRI) that suggested Wernicke’s encephalopathy (WE). She was treated with high-dose IV thiamine. Following treatment, her symptoms fully resolved.

Case 2 is a 70-year-old woman with short bowel syndrome due to multiple small bowel resections in the context of hollow visceral myopathy. She was on HPN seven days a week and had a venting gastrostomy (G) tube. She regularly vented significant and variable amounts of fluids depending on consumption of certain foods and liquids. During the IV multivitamin shortage, she adhered to medical recommendations and took an oral multivitamin (compliant). However, the supplement selected by the patient contained most B vitamins but lacked thiamine (Table S1). Three months into the shortage, she developed generalized weakness, confusion, and diplopia. An MRI revealed subtle FLAIR signal changes in the medial thalami, periaqueductal gray, and dorsal midbrain/mammillary bodies. Given her symptoms and MRI findings, she was diagnosed with WE. She was treated with high-dose IV thiamine. Her symptoms fully resolved before hospital discharge.

Case 3 is a 56-year-old woman on HPN for a malignant bowel obstruction due to metastatic ovarian cancer requiring HPN seven days a week. She had minimal oral intake, with most of it being vented through a G tube. During the IV multivitamin shortage, she reported taking an oral liquid multivitamin three times a week, therefore non-compliant (Table S1). Also, it was unclear how much was absorbed. Around three months after the shortage began, she developed vertigo, gait instability, and diplopia. She was hospitalized for five days, and her symptoms were initially suspected to be because of WE. She was treated with high-dose IV thiamine, but her symptoms did not improve. Further investigation with a brain MRI revealed new metastases in her left cerebellum, which better explained her neurologic symptoms. Given the findings, she was scheduled for palliative radiation therapy.

## 4. Discussion

In our center, during an IV multivitamin shortage, only 56% of patients were compliant taking oral multivitamin supplements for at least 5 days a week. Patients who complied to these recommendations were older and had longer HPN duration. Reasons for non-compliance included inconsistent use, being forgetful, and financial difficulties in affording oral supplements. There were no significant differences in pre- and post-shortage bloodwork, but there was a significant decrease in bicarbonate in the compliant group and two cases of Wernicke’s encephalopathy among high-risk patients.

The complete absence of IV multivitamins in HPN prescriptions due to backlog issues has been an ongoing problem [9,10]. During the IV multivitamin shortage, the recent ASPEN 2024 guideline suggests switching to oral or enteral multivitamins during a shortage, while patients with severe malabsorption syndromes may need double doses of oral multivitamins per day [17]. Guidelines also suggest that patients with malabsorption may benefit from alternative oral formulations, such as liquid, sublingual, chewable, or gummy forms. However, it is important to note that some oral liquid products contain sorbitol, which may lead to gastrointestinal side effects, including diarrhea [17]. Variability among multivitamin brands and inconsistent vitamin concentrations made it difficult to ensure adequate intakes for all patients [14,16]. Absorption rates were uncertain, especially in patients with short bowel syndrome, vomiting, or venting G tubes [18]. Those with bowel obstructions or severe dysmotility were particularly vulnerable, as they often refused or vented oral intake, leading to poor or no absorption [19]. These differences complicate interpretation, as patient absorption rates and supplement types vary. Therefore, IV multivitamin infusion is essential for most HPN patients, especially those dependent on HPN for five to seven days per week with minimal oral intake. Vitamin deficiencies developed at different time points depending on factors such as solubility, tissue storage, patient needs, dietary intake, and gastrointestinal anatomy [20]. In our study, no significant difference was observed between those who took oral multivitamins and those who did not, likely due to the long half-life of fat-soluble vitamins. Water-soluble vitamins, particularly thiamine, are more prone to early deficiencies [21]. Clinical deficiencies were difficult to detect since vitamin B levels, including thiamine, were not routinely monitored, symptoms were nonspecific, and tissue storage was limited. Additionally, our study observed reduced bicarbonate levels in non-compliant patients, suggesting a potential link to subclinical thiamine insufficiency. Future studies should consider incorporating direct biomarkers, such as erythrocyte transketolase activity (ETKA) and the ETKA activation coefficient, to assess thiamine status more accurately. These measures can help identify subclinical deficiencies and guide appropriate interventions.

Thiamine is essential for carbohydrate metabolism, nucleic acid synthesis, ATP production, and neurotransmitter formation [15]. Deficiency can develop rapidly, leading to acute neurological conditions such as WE, characterized by ocular signs, altered consciousness, and ataxia [22]. Ocular signs include nystagmus, bilateral lateral rectus palsies, and conjugate gaze palsies [23]. Previous multivitamin shortages have frequently led to thiamine deficiency, as reported by several HPN centers [11]. In 1989, the Centers for Disease Control and Prevention (CDC) documented three deaths from refractory lactic acidosis during a PN multivitamin shortage, with brain autopsies confirming acute thiamine deficiency [24]. Similarly, in 1997, the CDC reported three more cases of lactic acidosis caused by thiamine deficiency. Patients who went 1–2 months without oral or IV multivitamins were hospitalized with WE [7]. Pediatric patients also experienced severe thiamine deficiency during IV multivitamin shortages, with diagnoses confirmed by MRI [25]. Some patients developed WE despite taking oral multivitamins, as persistent vomiting prevented adequate absorption [11]. In this study, two cases of WE were reported, both involving patients who used oral multivitamins but likely did not absorb enough due to gastrointestinal losses [26]. The variability in multivitamin formulations, some containing thiamine and others not, along with factors such as vomiting and venting via G-tubes further compromised micronutrient intake and absorption, increasing the risk of thiamine and other vitamin deficiencies [27,28]. Serum thiamine levels are not routinely measured, as they do not always reflect cerebrospinal fluid levels, making WE diagnosis challenging since normal serum levels do not rule out the diagnosis of WE [29,30]. MRI findings in chronic cases may occur too late to prevent permanent structural damage [29,30]. Based on this experience, we recommend regular IV thiamine supplementation tailored to HPN patients at risk, such as those who rely on HPN for at least five days a week and experience high output from venting G tube, fistula, or vomiting during the IV multivitamin shortage. This is especially important if a multivitamin shortage lasts more than one or two months but can represent logistical challenges to arrange at a hospital-based infusion unit for ambulatory HPN patients. This is the challenge we encountered when attempting to organize hospital-based infusion because of lack of space. This underscores the need for planning optimal thiamine supplementation strategies in case of future shortages, including reserving space for in-hospital infusion in HPN patients at high risks.

Generally, ASPEN and The European Society for Clinical Nutrition and Metabolism (ESPEN) guidelines recommend monitoring vitamins and trace elements in HPN patients every three to twelve months, as deficiencies typically develop slowly [31,32]. However, these guidelines do not include routine monitoring of vitamin B levels except for vitamin B12 and folate [31,32]. Our study suggests that standard or more frequent monitoring may not be sufficient during a multivitamin shortage, as evidenced by the lack of differences in laboratory results before and after the shortage, likely due to either tissue storage issues or long half-lives of certain vitamins. Our study also highlights the limitations of relying on oral supplements as substitutes for IV multivitamins during shortages and the need for proactive strategies to manage supply disruptions. Since thiamine is absorbed in the small intestine, and its oral absorption does not exceed 4.5 mg even when high doses are administered, we believe that adding IV thiamine to HPN formulations may be a viable solution for the aforementioned high-risk patients [33]. Given the limited number of IV multivitamin manufacturers, similar shortages may occur in the future. HPN patients are a heterogeneous group, requiring individualized strategies rather than a one-size-fits-all protocol [34]. Close monitoring should assess not only deficiency symptoms and bloodwork but also patient compliance with oral supplements. Addressing oral supplementation issues requires selecting appropriate multivitamin brands, providing financial support, and considering outpatient IV thiamine infusions for high-risk patients. These measures will help mitigate risks associated with future shortages and improve patient outcomes.

There were limitations to our assessment. Our sample size was restricted due to the strict inclusion criteria, as we aimed to specifically reflect PN-dependent patients. While this focus improves the internal validity for this vulnerable group, it limits the generalizability of our findings. The experiences of these 25 patients may not represent the broader population affected by IV multivitamin shortage, including those with partial dependence on PN or receiving care in different healthcare settings across Ontario or Canada. To improve generalizability, future studies should include a larger cohort of patients requiring PN, including hospitalized individuals who were also affected by the supplement shortage. Expanding the sample to include patients from multiple centers would enhance the representativeness of the findings and better reflect standard clinical practice.

Furthermore, the survey was retrospective with open-ended questions, and patients may have underreported their symptoms. It is possible that more patients had mild cases of thiamine deficiency but were not diagnosed if they did not seek medical attention. Further studies should evaluate the adequacy of oral multivitamins in maintaining vitamin B levels, including thiamine, during shortages. They should also identify specific dosages of oral thiamine supplements for HPN patients, particularly those with malabsorption, when IV thiamine is not feasible. Despite the limitations, this study provides initial insights into a clinically relevant issue in a highly vulnerable patient population and underscores the need for proactive management strategies during micronutrient shortages.

## 5. Conclusions

Oral multivitamin supplementation should be implemented as soon as an IV multivitamin shortage occurs to ensure micronutrient adequacy. Overall, this study suggests that identifying patients at risk, monitoring symptoms of vitamin deficiencies, and assessing compliance for oral multivitamin as per the recommendation are important. Patients who rely on HPN should be prioritized due to their minimal absorptive capacities and hence increased risk of vitamin deficiency. The low percentage of oral multivitamin adherence also calls for a greater need for regular follow-ups to ensure greater patient compliance with medical instruction.

## Figures and Tables

**Table 1:** Baseline Patient Characteristics in Cohort.

Variables	Overall (*n* = 25)	Vitamins Taken (*n* = 14)	Vitamins Not Taken (*n* = 11)	*p*-Value
Age (years)	57 (42, 68)	64 (55, 70)	43 (21, 67)	0.037
Female	17 (68%)	12 (85.71%)	5 (45.45%)	0.081
Height (cm)	163 (158, 172)	164 (162, 169.5)	159 (152, 175)	0.57
Weight (kg)	55.5 (48, 68.2)	61.6 (53, 74.4)	48.9 (40.6, 68.2)	0.12
BMI (kg/m^2^)	20.7 (18.7, 23.6)	21.35 (20.2, 24.8)	187 (16, 21.6)	0.08
Indication for PN				
SBS	15 (60%)	8 (57.14%)	7 (63.64%)	0.63
GI dysmotility	5 (20%)	4 (28.57%)	1 (9.09%)	
Malignancy	5 (20%)	2 (14.29%)	3 (27.27%)	
Some oral intakes	17 (68%)	10 (71.4%)	7 (63.63%)	0.86
Use of venting G tube	2 (8%)	1 (7.14%)	1 (9.09%)	1.00
TC (kcal/d)	1542 (1368, 1792)	1516 (1400, 1735)	1545 (1334, 1800)	0.87
Protein (g/d)	75 (60, 95)	75 (65, 95)	75 (55, 95)	0.51
Lipid (g/d)	50 (45, 55)	50 (45, 55)	45 (40, 60)	0.36
Dextrose (g/d)	250 (200, 260)	250 (200, 260)	225 (210, 280)	0.91
PN (d/week)	7 (6, 7)	7 (6, 7)	7 (7, 7)	0.07
PN duration (months)	42 (7, 56)	52.5 (20, 83)	9 (6, 42)	0.02
PN volume (mL)	1800 (1680, 2400)	2000 (1650, 2580)	1800 (1710, 2200)	0.39

Values presented as median (1st and 3rd quartile) or *n* (%), as appropriate. Abbreviations: BMI: body mass index; G tube: gastrostomy tube; GI: gastrointestinal; PN: parenteral nutrition; SBS: short bowel syndrome; TC: total calories.

**Table 2:** Effect of Multivitamin Status on Clinical Outcomes.

	Overall (*n* = 25)	Vitamins Taken (*n* = 14)	Vitamins Not Taken (*n* = 11)	*p*-Value
Symptom reported	17 (68%)	9 (64.29%)	8 (72.73%)	1.00
Hospitalization	3 (12%)	2 (14.29%)	1 (9.09%)	1.00
Diagnosed deficiency	2 (8%)	2 (14.29%)	0 (0%)	0.50

Values presented as *n* (%).

**Table 3:** Biochemical and Vitamin Outcomes Before and After Shortage.

	Baseline Level					After Shortage Concluded					Change				
	N	Vitamins Taken	N	Not Taken	*p*	N	Vitamins Taken	N	Not Taken	*p*	N	Vitamins Taken	N	Not Taken	*p*
Glucose (mmol/L)	14	5.15 (4.8, 5.7)	11	6.0 (5.3, 6.3)	0.03	13	5.90 (5.20, 6.40)	11	5.50 (5.20, 6.40)	0.77	13	0.4 (−0.5, 2.3)	11	−0.2 (−0.80, 1.10)	0.3
BUN (mmol/L)	14	8.40 (5.6, 11.1)	9	7.70 (6.3, 10.5)	0.75	13	6.30 (5.20, 9.70)	8	8.00 (5.25, 11.70)	0.83	13	−0.3 (−1.1, 0.1)	8	−0.65 (−1.70, 1.30)	0.64
Creatinine (μmol/L)	14	87 (61, 122)	11	60 (57, 81)	0.14	13	88 (61, 107)	11	61 (48, 86)	0.12	13	−4 (−22, 10)	11	4 (−12, 9)	0.28
Sodium (mmol/L)	14	139.5 (137, 141)	11	136 (135, 138)	0.05	13	140 (136, 141)	11	135 (135, 137)	0.04	13	0 (−4, 2)	11	−1 (−2, 2)	0.82
Potassium (mmol/L)	14	3.95 (3.80, 4.30)	11	4.10 (3.80, 4.50)	0.89	13	3.90 (3.60, 4.60)	11	4.20 (3.80, 4.80)	0.41	13	−0.1 (−0.4, 0.4)	11	0.30 (0.10, 0.40)	0.31
Bicarbonate (mmol/L)	11	23 (20, 27)	8	25 (23.5, 28)	0.32	10	23.5 (20, 26)	8	24 (23, 24.5)	0.93	10	0 (−0.9, 1)	7	−2 (−8, −1)	0.04
Chloride (mmol/L)	14	105 (101, 107)	11	105 (103, 106)	0.72	13	103 (102, 107)	11	105 (102, 107)	0.95	13	0 (−3, 3)	11	−1 (−4, 1)	0.6
Calcium (mmol/L)	13	2.26 (2.21, 2.51)	11	2.22 (2.14, 2.33)	0.47	13	2.36 (2.22, 2.47)	11	2.35 (2.12, 2.42)	0.79	12	0.02 (−0.16, 0.16)	11	0.05 (−0.05, 0.18)	0.88
Mg (mmol/L)	14	0.82 (0.78, 0.93)	11	0.82 (0.76, 0.96)	0.91	13	0.89 (0.82, 0.91)	11	0.80 (0.76, 0.90)	0.27	13	−0.01 (−0.12, 0.08)	11	0.01 (−0.13, 0.07)	0.75
Phosphate (mmol/L)	14	1.18 (1.03, 1.31)	11	1.12 (0.93, 1.36)	0.48	13	1.10 (0.97, 1.18)	11	1.23 (1.03, 1.34)	0.16	13	−0.06 (−0.24, 0.05)	11	0.10 (−0.12, 0.28)	0.12
ALT (U/L)	14	22 (16, 38)	11	20 (12, 49)	0.83	13	27 (14, 31)	11	12 (10, 24)	0.1	13	−5 (−13, 3)	11	−6 (−39, −2)	0.21
ALP (U/L)	14	91 (74, 147)	11	111 (79, 407)	0.6	13	117 (92, 140)	11	105 (72, 232)	0.98	13	7 (−4, 14)	11	3 (−53, 82)	0.98
GGT (U/L)	14	39 (14, 84)	9	33 (23, 150)	0.47	12	42.5 (16, 102)	9	29 (22, 64)	0.97	12	−0.5 (−16.5, 2.5)	9	−1 (−25, 5)	0.67
Bilirubin (μmol/L)	14	7.0 (4.0, 12.0)	11	6.0 (5.0, 9.0)	0.87	13	8.0 (4.0, 13.0)	11	7.0 (5.0, 11.0)	0.73	13	0.0 (−1.0, 1.0)	11	1.00 (−6.00, 4.00)	0.66
Albumin (g/L)	14	37.0 (34.0, 39.0)	11	33.0 (26.0, 42.0)	0.46	13	39.0 (34.0, 42.0)	11	32.0 (31.0, 39.0)	0.28	13	1.6 (−4.0, 3.0)	11	0.0 (−4.0, 7.0)	1
Vitamin A (μmol/L)	10	1.75 (1.00, 2.10)	6	1.50 (1.30, 2.20)	0.62	6	1.90 (1.20, 2.80)	5	1.90 (1.60, 2.30)	1	6	0.1 (−0.10, 0.2)	4	−0.00 (−0.60, 0.90)	1
Vitamin D (μmol/L)	11	58.0 (35.0, 73.0)	6	38.5 (21.0, 57.2)	0.08	8	62.5 (45.5, 85.8)	5	40.4 (31.0, 53.0)	0.24	7	0.0 (−1.0, 9.7)	4	4.5 (−2.5, 14.2)	0.57
PTH (pmol/L)	10	5.95 (3.40, 9.30)	4	2.40 (1.85, 4.35)	0.07	4	7.25 (5.35, 8.25)	3	5.40 (1.80, 6.20)	0.16	3	2.10 (0.30, 2.40)	2	1.20 (−0.40, 2.80)	1
Vitamin E (μmol/L)	1	33 (33, 33)	2	33 (32, 34)	N/A	5	39.0 (36.0, 41.7)	5	31.9 (28.0, 33.5)	0.12	0	NA	1	−4.0 (−4.0, −4.0)	N/A
INR	14	1.10 (1.00, 1.16)	10	1.05 (1.00, 1.20)	0.85	13	1.00 (1.00, 1.10)	9	1.20 (1.10, 1.20)	0.04	13	0.0 (−0.1, 0.0)	8	0.10 (0.00, 0.15)	0.03
B12 (pmol/L)	11	544 (352, 871)	8	574 (402.5, 762.5)	0.93	11	414 (363, 835)	7	465 (269, 598)	0.29	10	−143.5 (−213, −30)	7	−66 (−266, 1)	0.56
Hemoglobin (g/L)	14	115.5 (95, 121)	10	101.5 (82, 125)	0.56	13	122 (102, 128)	10	100.5 (88, 141)	0.57	13	2 (−8, 10)	10	2 (−16, 16)	0.93
Platelets (×10^9^/L)	14	182.0 (164.0, 291.0)	10	294.5 (215.0, 397.0)	0.04	13	181.0 (167.0, 280.0)	10	317.0 (251.0, 411.0)	0.05	13	−3.0 (−30.0, 38.0)	10	−1.0 (−93.0, 69.0)	1
WBC (×10^9^/L)	14	0 (0, 0)	10	0 (0, 0)	0.24	14	0 (0, 0)	10	0 (0, 0)	1	14	0 (0, 0)	9	0 (0, 0)	0.21

Values presented as median (1st and 3rd quartile). Abbreviations: ALT: alanine aminotransferase; ALP: alkaline phosphatase; BUN: blood urea nitrogen; GGT: gamma-glutamyl transferase; INR: international normalized ratio; N/A: non-applicable; PTH: parathyroid hormone; WBC: white blood cells.

**Table 4:** A Case Series of Patients Who Required Hospitalization.

Variable	Patient No. 1	Patient No. 2	Patient No. 3
Age (years)	59	70	56
Sex	Female	Female	Female
Weight (kg)	67.1	68.4	48.5
Body Mass Index (kg/m^2^)	25.25	25.74	20.88
Indication for PN	High output entero-vaginal fistula	Short bowel syndrome in the context of hollow visceral myopathy	Malignant bowel obstruction secondary to metastatic ovarian cancer
Oral diet	No	Minimal oral diet	No
Oral multivitamin	Centrum	Vitafusion	Nature’s Bounty Liquid B Complex
Use of venting tube	None	2–2.5 L/d	Substantial
Total calories (kcal/d)	1920	1252	1654
Protein (g/d)	100	60	80
Dextrose (g/d)	280	180	260
PN frequency (d/week)	7	7	7
Add-ins	Octreotide 300 mcg	Famotidine 20 mg	Famotidine 40 mg
Precipitate	Severe nausea and vomiting	None	None
Neurological symptoms	Dizziness, double vision and weakness	Diplopia, declining mobility and weakness, and dizziness	Vertigo, gait instability, and diplopia
MRI results	Abnormal FLAIR signal observed in the dorsal medial thalami, around the third ventricle, tectal plate, and periaqueductal gray matter, accompanied by subtle increased signal intensity and facilitated diffusion on diffusion-weighted imaging	Subtle increased FLAIR signal noted in the medial thalami, periaqueductal gray, with subtle enhancement through the dorsal midbrain/memory bodies	Interval growth of left inferior cerebellar lesion. Normalization of signal abnormalities dorsomedially in the thalami with equivocal persistent increased signal in the peri aqueductal matter

## Data Availability

The data are not publicly available due to privacy concerns.

## Supplementary Materials

The following supporting information can be downloaded at https://www.mdpi.com/article/10.3390/nu17091500/s1. Table S1: Intravenous multi-12 vs. over the counter oral vitamin content; Data S1: Survey questionnaire used in study; Table S2: Effect of multivitamin status on anthropometric outcomes after multivitamin shortage concluded and the change of anthropometric outcomes.

## Abbreviations

ASPEN	American Society for Parenteral and Enteral Nutrition
CDC	Centers for Disease Control and Prevention
DRIs	Dietary reference intakes
ESPEN	European Society for Clinical Nutrition and Metabolism
ETKA	Erythrocyte transketolase activity
G-tube	Gastrostomy tube
HPN	Home parenteral nutrition
IV	Intravenous
MRI	Magnetic resonance imaging
PN	Parenteral nutrition
WE	Wernicke’s encephalopathy
