# Supplementary material for: The Impact of a Prolonged Multivitamin Shortage on Home Parenteral Nutrition Patients: A Single-Center Retrospective Cohort Study with Case Reports of Wernicke’s Encephalopathy
Source: Nutrients. 2025 Apr 29;17(9):1500. doi: 10.3390/nu17091500 (PMC12073724; doi:10.3390/nu17091500)
Supplement: Supplementary file 1 [file nutrients-17-01500-s001.zip › nutrients-3598528-supplementary.pdf]

Supplementary Materials

Table S1: Intravenous Multi-12 VS Over the Counter Oral Vitamin Content

Ingredient	PN Home & long term recommendation	Multi-12 mixed solution	DRI per day for age 31-70 years	Jamieson Multivitamin (n=2)	Vitafusion MultiVites (n=2)	Nature's Bounty (n=1)	Centrum (n=2)	Webber Naturals (n=2)	Nature's Bounty Liquid B Complex (n=1)
Dose		10 ml		1 caplet/d	2 gummies/d	1 gummy/d	1 tablet/d	1 tablet/d	1 mL 3 times/week
Vitamin A (mcg)	800-1100	1000	700-900	300	450	750	300	1510	0
Beta-carotene (mcg)				100	0	0	600	0	0
Vitamin B1 (mg)	2.5	6	1.1-1.2	3.85	0	0	3.85	1.5	0
Vitamin B2 (mg)	3.6	3.6	1.1-1.3	3.85	0	0	3.85	1.7	2
Vitamin B3 (mg)	40	40	11-16	14	8	0	14	20	20
Vitamin B5 (mg)	15	15	5	11	0	0	11	0	30
Vitamin B6 (mg)	4	6	1.5-1.7	5	2	2	5	2	2
Vitamin B 7 Biotin (mcg)	60	60	30 (AI)	45	0	0	45	0	0
Vitamin B12 (mcg)	5	5	2.4	12	7.2	9	12.6	6	1200
Vitamin C (mg)	100-200	200	75-90	150	0	30	150	60	0
Vitamin D (IU)	200	200	600-800	800	1000	1000	800	400	0
Vitamin E (mg)	≥ 9	10	15	18.76	18.8	13.5	12.8	4.5	0
Vitamin K1 (mcg)	150	NA	90-120	90	0	0	20	0	0
Folate (mcg)	400	600	400 DFE	600	400	666	400	100	0
Chromium (mcg)	10-15	NA	20-35	25	35	0	25	0	0
Copper (mcg)	300-500	NA	900	900	0	0	0	0	0
Iodine (mcg)	130	NA	150	150	0	0	150	0	0
Iron (mg)	1	NA	8	7.5	0	0	7.5	0	0
Manganese (mg)	55	NA	1.8-2.3	5	0	0	0.9	0	0
Selenium (mcg)	60-100	NA	55	55	0	0	55	0	0
Zinc (mg)	3-5	NA	8-11	8	2.2	2.5	8	0	0

Data S1: Survey Questionnaire used in Study

Dear HTPN Patient,

We are contacting you to inform you that the multivitamin shortage has ended, and we will be resuming your multivitamin in your TPN bags. We are hoping to better assess patient's experience during this shortage. Please answer the following questions:

1. How many days of TPN do you currently require?
2. During the shortage, did you take an oral multivitamin? If so, what brand did you use?
   ☐ Yes ☐ No
   If yes, how many days per week did you take multivitamins?
   If yes, how many pills/dosages did you take of the multivitamin?
3. If you did take an oral multivitamin, did you have any NEW symptoms or side effects related to taking the ORAL multivitamin? Please describe.
4. If you did NOT take an oral multivitamin, did you have any NEW symptoms during the time without IV Multivitamin? Please describe.

Table S2: Effect of Multivitamin Status on Anthropometric Outcomes After Multivitamin Shortage Concluded and the Change of Anthropometric Outcomes

	N	Overall (n=25)	N	Vitamins Taken (n=14)	N	Vitamins Not Taken (11)	P-value	N	Overall (n=25)	N	Vitamins Taken (n=14)	N	Vitamins Not Taken (11)	P-value
	Anthropometrics after shortage							Change in Anthropometrics						
Weight (kg)	21	61.00 (49.10, 68.00)	11	61.00 (50.90, 68.40)	10	57.90 (48.50, 68.00)	0.7781	21	0.90 (-2.50, 3.10)	10	0.30 (-3.50, 2.40)	10	2.70 (-2.40, 8.50)	0.1130
Body Mass Index (kg/m^2)	21	21.70 (19.80, 23.80)	11	22.10 (20.50, 24.70)	10	20.50 (18.00, 23.80)	0.4808	21	0.30 (-0.90, 1.44)	11	0.20 (-1.30, 0.90)	10	1.02 (-0.90, 3.20)	0.1487
	Parenteral Nutrition after shortage							Change in Parenteral composition						
Amino Acids (kcal/day)	25	300.00 (240.00, 380.00)	14	300.00 (260.00, 380.00)	11	300.00 (220.00, 380.00)	0.3641	25	0.00 (0.00, 0.00)	14	0.00 (0.00, 0.00)	11	0.00 (0.00, 0.00)	0.2593
Lipid (kcal/day)	25	500.00 (450.00, 550.00)	14	500.00 (450.00, 550.00)	11	450.00 (400.00, 600.00)	0.3322	25	0.00 (0.00, 0.00)	14	0.00 (0.00, 0.00)	11	0.00 (0.00, 0.00)	0.2593
Dextrose (kcal/day)	25	850.00 (680.00, 884.00)	14	850.00 (680.00, 884.00)	11	765.00 (714.00, 952.00)	0.9124	25	0.00 (0.00, 0.00)	14	0.00 (0.00, 0.00)	11	0.00 (0.00, 0.00)	0.2593

Values presented as median (1st, 3rd quartile) or n (%), as appropriate.
